# BrEPS: a flexible and automatic protocol to compute enzyme-specific sequence profiles for functional annotation

**DOI:** 10.1186/1471-2105-11-589

**Published:** 2010-12-01

**Authors:** C Bannert, A Welfle, C aus dem Spring, D Schomburg

**Affiliations:** 1 Dept. of Bioinformatics and Biochemistry, Technische Universität Braunschweig, Langer Kamp 19b, 38106 Braunschweig, Germany

## Abstract

**Background:**

Models for the simulation of metabolic networks require the accurate prediction of enzyme function. Based on a genomic sequence, enzymatic functions of gene products are today mainly predicted by sequence database searching and operon analysis. Other methods can support these techniques: We have developed an automatic method "BrEPS" that creates highly specific sequence patterns for the functional annotation of enzymes.

**Results:**

The enzymes in the UniprotKB are identified and their sequences compared against each other with BLAST. The enzymes are then clustered into a number of trees, where each tree node is associated with a set of EC-numbers. The enzyme sequences in the tree nodes are aligned with ClustalW. The conserved columns of the resulting multiple alignments are used to construct sequence patterns. In the last step, we verify the quality of the patterns by computing their specificity. Patterns with low specificity are omitted and recomputed further down in the tree. The final high-quality patterns can be used for functional annotation. We ran our protocol on a recent Swiss-Prot release and show statistics, as well as a comparison to PRIAM, a probabilistic method that is also specialized on the functional annotation of enzymes. We determine the amount of true positive annotations for five common microorganisms with data from BRENDA and AMENDA serving as standard of truth. BrEPS is almost on par with PRIAM, a fact which we discuss in the context of five manually investigated cases.

**Conclusions:**

Our protocol computes highly specific sequence patterns that can be used to support the functional annotation of enzymes. The main advantages of our method are that it is automatic and unsupervised, and quite fast once the patterns are evaluated. The results show that BrEPS can be a valuable addition to the reconstruction of metabolic networks.

## Background

The functional annotation of newly sequenced genes is a classic problem in computational biology. Even though dozens of annotation protocols exist, many of them are general purpose, not tailored to a special application. One such application is the reconstruction of metabolic or regulatory networks in systems biology. To accurately reconstruct the metabolic network of a given organism, it is necessary to precisely determine its enzyme repertoire. The enzymes in the genome have to be found and their function (defined by EC-numbers) has to be determined.

Sequence database searching is a standard method for functional annotation that is also used in the reconstruction of metabolic networks. A database of known sequence targets is searched with the genes of the organism in question. If there is sufficient similarity between a query and a target, the function of the target gene can be inferred to the query. Our group uses such an approach ("Enzyme Detector", manuscript in preparation) to identify the enzymes of a given organism. The results of a BLAST-dependent [1] search are combined with other results to predict the presence of an enzyme. In many cases, sequence-similarity based methods, however, do not allow an unambiguous decision. One of the reasons is given by the fact that many enzymes have similar sequences, even though their biochemical function differs [2]. Another source of errors are multidomain proteins. A protein domain is usually defined as a structural and functional unit of structure, folding independently of the rest of the protein chain (see e.g. [3]). Many proteins are composed of several domains, and some domains are frequently used in different combinations [4]. This often complicates functional annotation.

A classical approach in functional annotation consists of the determination of sequence similarity, followed by sequence clustering into protein families, and the representation of these families by family-specific sequence profiles or patterns (for an overview, see reviews [5-8]). Some of these methods include an intermediate step that copes with multidomain proteins. We shortly introduce a number of these methods with emphasis on those using patterns or not being covered by [5-8].

Sequence database searching is often the first step in the annotation protocol, used to determine the similarity or homology of the input sequences. Once the homologous sequences are merged into clusters or families, their common properties are captured in some kind of profile. The construction of sequence patterns is one way to do that. Sequence patterns can be deterministic or probabilistic [9]. Deterministic patterns work like a filter or a fingerprint, specifying a set of possible subsequences that will match the pattern or not. They allow a clear "yes" or "no" decision on the question if a given sequence matches the profile, which can be advantageous in some research contexts. Despite their lack of flexibility, deterministic sequence patterns have been used in many projects [5].

Some approaches for pattern or motif recognition do not include an alignment strategy, PRATT [10] and CASTOR [11] are two examples. They require sets of unaligned sequences as input, which usually need to be related to construct biologically meaningful patterns. PRATT allows to construct patterns with user-defined properties, e.g. the patterns may contain gaps of variable length or allow ambiguous positions. CASTOR is a clustering protocol using sequence patterns to recursively divide the input sequences. Statistically relevant patterns are further refined in each step. CASTOR is an example of an automatic and unsupervised method that does not require manual intervention, as long as the input sequences are known to be related. Prosite [12] was initially a repository of manually curated patterns. Today, the Prosite team also uses automatic and semi-automatic techniques, and many patterns are based on HMM profiles [12].

The methods we have introduced so far are general, not being specialized in a certain class of protein. In contrast, PRIAM [13] represents an approach restricted to enzymes. PRIAM first collects all enzyme entries from the ENZYME database [14] and retrieves the sequences for each given enzyme from SwissProt. Then it identifies the conserved 'modules' in each group of sequences with PSI-BLAST [15]. These modules are converted into position-specific scoring matrices (PSSMs), which can be used for functional annotation. We are currently not aware of another method that is specialized on the functional annotation of enzymes.

We present an unsupervised and automatic method for the functional annotation of enzymes, to support the reconstruction of metabolic networks. Its name is BrEPS, short for "*Braunschweig Enzyme Pattern Search*". Given a small number of parameters, it automatically creates sequence patterns from groups of enzyme sequences. Different from PRIAM, BrEPS aims at defining minimal sequence patterns for one or more EC numbers.

**Figure 1:**
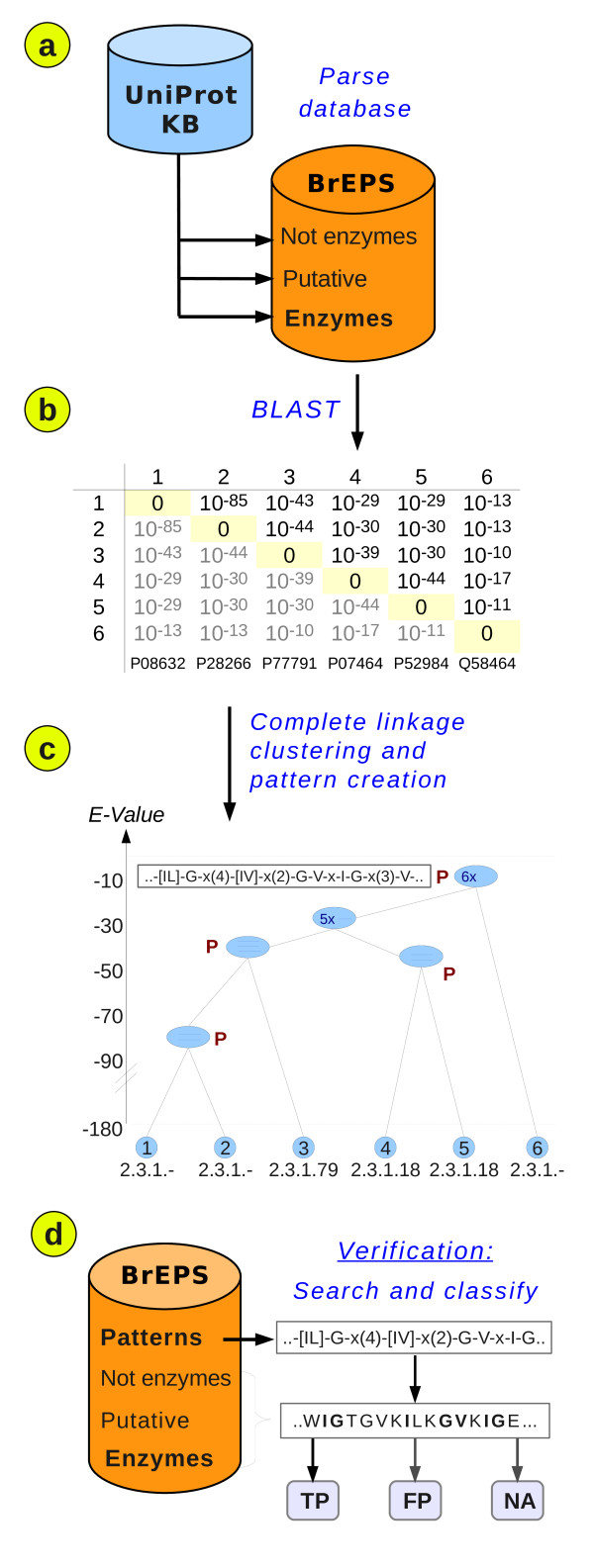
**Breps Protocol: Outline**. Outline of the BrEPS protocol, which comprises of four different steps. See the main text for a detailed description.

In the first step the enzyme sequences in the Swiss-Prot part of the UniprotKB are identified and sorted into three categories. The enzyme sequences are then compared by an all-vs.-all BLAST. The resulting E-Values of the computed BLAST alignments are used as distance measure in a Complete Linkage clustering of the enzymes. The sequences in the clusters of the created trees are aligned with ClustalW [16]. The conserved positions in the multiple alignment are converted into a sequence pattern corresponding to enzyme function. Finally, we estimate the fitness of these patterns by searching the BrEPS database for True- and False positive hits. See Figure 1 for an overview.

## Results

### Preprocessing

We retrieve the Swiss-Prot part of the UniProtKB and parse their description ("DE") lines. Depending on the presence of EC numbers, certain keywords and sequence length we sort the Swiss-Prot entries into three groups: Entries with an EC number are considered as *enzymes*, if their length is between 100 and 7000 amino acids. No complete enzyme in BRENDA [17] is shorter than 100 positions and only 0.1% of the Swiss-Prot sequences in the UniProtKB are longer than 7000 positions. The enzymes make up the first group (Figure 1a).

Fragments, regulatory domains without enzymatic activity, or activation peptides are identified by keywords like "putative", "hypothetical" or "fragment". These entries are put into the second, *putative *group. We assume the function of these proteins to be unknown, they need not even be real expressed proteins. All remaining proteins, which have neither an EC number nor one of the aforementioned keywords, are moved into the third group, *Proteins without enzymatic activity *(see also Figure 1a):

1. Enzymes.

2. Putative proteins/fragments with unknown function.

3. Proteins without enzymatic activity

Our protocol proceeds with the enzymes in group one. The proteins in group two and three are only used in the verification of the final patterns (see "Pattern Verification"). To reduce the computational overhead of the protocol, redundant entries in the source database are excluded from subsequent steps. In group two and three, we define two entries to be redundant if their sequence is identical. This is different in group one, where every entry is associated with one or more EC numbers.

Let *ec*(*X*) be a function that returns the set of EC numbers associated with an object *X*. For example, assume this object to be an enzyme *q*, with *ec*(*q*) = {'1.2.3.4', '2.2.27.-'}. Given two enzymes *q, r: *we call *r *redundant to *q *if and only if

a) the sequences of *q *and *r *are identical and

b.1) *ec*(*q*) = *ec*(*r*), i.e. they are annotated with the same set of EC numbers or

b.2) *ec*(*r*) is a subset of *ec*(*q*).

In the following step the enzymes in our database are checked for changes of their EC numbers. For example, a given EC number may have been deleted by the IUBMB or replaced by a new one. Transferred EC numbers are updated and entries with deleted EC numbers are removed from the database.

Then, all enzyme sequences are submitted to a BLAST all-vs-all comparison. Low complexity sequences are removed in this step, by applying the SEG filter [18]. The start and end indices of the remaining pairwise BLAST alignments are stored in our database. We also store the associated BLAST E-Value. Alignments covering less than 50 positions and those with an E-Value higher than 1·10^-3 ^are discarded (Figure 1b).

### Clustering of the enzyme sequences

After the preprocessing, the enzymes are clustered by Complete Linkage (CL) clustering (also known as maximum linkage clustering). CL clustering is a method for agglomerative clustering. Given a set of pairwise distances between the clustered objects, the pair of objects with the smallest distance will be merged into a new cluster in each iteration of the algorithm. Then the distances of all nodes to the newly formed cluster have to be recomputed. In CL clustering, the recomputed distance between the newly formed cluster and another object will always be the maximum of all available distances to that object.

Agglomerative clustering usually results in a single tree. Each enzyme in our database can be thought of being a leaf at the bottom of that tree. The distance measure between the leafs are the BLAST E-Values from the all-against all comparison described above. Since we discard similarities higher BLAST E-Values than 1·10^-3^, the clustering can only proceed until this E-Value has been reached. One can think of the complete linkage tree being cut horizontally at an E-Value of 1·10^-3^. Our clustering procedure therefore results in many trees that are written into an SQL database. Every tree node is associated with the BLAST E-Value of the last merge event with another leaf or node (Figure 1c).

The BLAST score *s *can be asymmetric with respect to the order of query *i *and target *j*: *s*(*i*, *j*) ≠ *s*(*j*, *i*). Therefore sometimes two different distances are calculated between a pair of enzymes. In this case, we keep the higher E-Value (indicating less sequence similarity) as clustering distance, which corresponds to a symmetrification of the similarity matrix. This also ensures that enzymes with the same domain composition will cluster with a higher priority than single domain enzymes with multidomain enzymes that contain this domain. In spirit, this is somewhat similar to choosing the strongly connected components in the directed graph used in [19] as clusters.

A few enzymes are not very similar enough to any other enzyme in our data set. They generate only a few or no BLAST hits at all, and are therefore not assigned to any tree. We refer to them as unclustered sequences. Each unclustered sequence is put in an artificial tree that has one node.

### Pattern computation

The trees in the database represent groups of similar enzyme sequences. Tree nodes with small E-Values are likely to contain a family of evolutionary related enzymes. We conserve a part of the evolutionary information in the family by creating a sequence pattern. If the pattern matches a query sequence, the biochemical function of the query can be inferred from the family.

It is not necessary to compute a pattern for every node in the tree because the set of associated EC numbers does not always change when sequences are added or nodes are merged. If every node had a pattern, this would result in high runtimes when searching a query sequence with unknown function. Any tree should however have at least one pattern, even if the tree contains only a few enzymes. Otherwise we could not be sure to detect input sequences that are already present in the BrEPS database. We therefore compute a pattern for any *qualified *node *N *if:

- *N *is the root OR

- *N *has fewer EC numbers than its father node

The trees are processed top-down starting with the root node. All sequences within a qualified node are subjected to a multiple alignment procedure using ClustalW. Highly conserved positions (marked with a star or colon) in the multiple alignment are transferred into a sequence pattern in ProSite [12] format (Figure 1c). There are however exceptions:

1. Because of the computational overhead, we do not treat nodes with 1000 or more sequences. These nodes usually contain sequences with low similarity and several EC numbers and result in short and therefore unspecific patterns.

2. If a pattern covers less than eight positions of a given input sequence, we discard the pattern because of its small significance.

Sometimes there is not a single conserved position in the alignment, even if we have aligned several hundreds of non-redundant enzyme sequences. It is therefore possible that a tree root is not represented by a pattern. It is even possible that a tree has no pattern at all, especially if there is only one EC number in the tree. We identify problematic trees by counting the percentage of enzymes that are represented by at least one pattern in the tree, %*pce *(pattern-covered enzymes). If %*pce *is below a predefined constant, we iteratively add (more) patterns to the tree: First, we identify all *candidate *nodes that have no pattern. Nodes that were already tried in earlier iterations but failed to achieve a valid pattern are excluded. The remaining nodes are sorted by descending E-Value, such that the worst E-Value(s) are at the top. Then, a pattern is computed for all nodes with identical E-Values. If at least one pattern could be kept, we check if %*pce *is still below our threshold. In that case, we continue with the next node(s), until either %*pce *is above the threshold, or there are no more nodes to try. After one tree is finished, we continue this procedure until all trees are processed.

The unclustered sequences we described in the "Clustering the enzymes" Section are also represented by "patterns". Their sequence serves as pattern, such that an exact match of a query sequence is possible. The information of these enzymes is therefore not completely lost.

### Pattern verification

Sensitivity and specificity of the patterns are important parameters to judge their quality. Unspecific pattern should not be used for functional annotation, except if there is no other source of information. We therefore search the proteins in our database with each of the computed patterns, including the proteins without enzymatic activity and the group of putative proteins. Redundant entries are excluded from the verification.

If a pattern matches an entry without enzymatic activity, we score a false positive hit (FP). If one of the entries in the "putative" group is matched, the hit gets a neutral status, because we cannot safely assign a result (NA). If our pattern is matching an enzyme, we have to compare its EC numbers with those of the matched sequence (Figure 1d). The result depends on the definition of these relationships. This is not trivial, because every pattern and every enzyme can be associated with more than one EC number. In addition, some of the EC numbers are incomplete, e.g. "1.1.-.-".

In our definition, an EC number connected to a pattern means that this pattern will find all enzymes with the same level of functional resolution. For example, a pattern *p *with *ec*(*p*) = {'1.1.2.-'} should identify all oxidoreductases that act on compounds carrying a CH-OH group, with a cytochrome as acceptor [17]. In this case the 4^th ^part of the EC-number is not specified in more detail. If *p *matched an enzyme *q *with *ec*(*q*) = {1.1.2.2}, we would score a true positive hit (TP). This is not the same if the two EC sets are swapped, i.e. the pattern-associated set *ec*(*p*) = {1.2.2.1} and the sequence-associated set *ec*(*q*) = {'1.2.2.-'}. In that case, the pattern is associated with cytochrome-acting mannitol dehydrogenases. Since we do not know if *q *has that function (it could also be an L- or D-lactate dehydrogenase), a decision is impossible (NA). Our pattern verification method is therefore asymmetric with respect to patterns and sequences.

If there is more than one EC number present in either the pattern or the enzyme sequence, we rank the relationships by the following rule: A TP gets precedence over a FP, and a FP gets precedence over a NA case. That means that we do not even need to evaluate a match between *p *and *q *any further as soon as we have found a TP. On the other hand, we can only assign NA if there is neither a TP nor a FP case present. Our scoring rules are best shown by the examples in Table 1.

**Table 1:** Pattern verification: Example cases

**No**.	Pattern ECs/*ec*(*p*)	Target ECs/*ec*(*q*)	Case
1.	*1.2.3.4*	*1.2.3.4*	TP
2.	*1.2.3.4*	*1.2.3.5*	FP
3.	*1.2.3.4*	*1.2.3.-*	NA
4.	*1.2.3.-*	*1.2.3.4*	TP
5.	*1.2.3.-*	*1.2.3.-*	TP
6.	*1.2.3.4, 2.6.1.2*	*1.2.3.4, 2.6.1.-*	TP
7.	*1.2.3.4, 2.6.1.2*	*1.2.3.5, 2.6.1.-*	FP
8.	*1.2.3.4, 2.6.1.2*	*1.2.3.4, 1.2.3.5, 2.6.1.-*	TP
9.	*1.-.-.-*	*1.2.3.4*	TP

Table 1 shows nine examples explaining the assignment of the True Positive (TP), False Positive (FP) and Undecided (NA) status to a pattern that is matching an enzyme in the database. A detailed explanation is provided in the main text.

**Figure 2:**
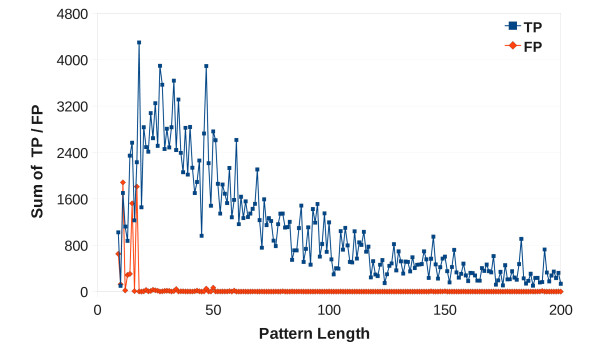
**Verification: True and False Positives at varying pattern lengths**. Figure 2 shows the sum of True Positive annotations (TP) and False Positive annotations (FP) at pattern lengths between 9 and 200. At small pattern lengths around ten, the sum of FPs is often close to the number of TPs. This is rapidly changing with increasing pattern length, most patterns of more than 50 positions are specific.

The results of the pattern verification are especially important, because the number of true positive (TP) and false positive hits (FP) are the main parameters to judge the quality of a pattern. Figure 2 shows the sum of TPs and FPs at pattern lengths up to 200. As expected, the number of FPs is decreasing with increasing pattern length. After the patterns have reached a length of 12 positions, the number of TPs is always above the number of FPs recognized by the pattern, which results in the high specificity of the patterns: About 99% of them have a specificity of more than 95%, only 0.2% of them have a specificity of less than 50%.

Two proteins with the same EC number can belong to different sequence families that need not to be closely related. Since our approach generates one or more patterns for each sequence family, determining the number of false negatives is not suitable as a quality measure in this setup. We have therefore not determined the number of false negatives (FNs).

### Statistics of the BrEPS database

We ran our protocol on the Swiss-Prot Release 57.6 (13th of October, 2009) of the UniProt Knowledge Base [20]. After parsing the data, we count 165,805 non-redundant enzymes and 228,406 non-redundant proteins without enzymatic activity. The group with the putative proteins and the fragments contains 33,769 non-r entries.

The enzyme sequences are clustered into 5,414 trees, including 794 single-Node trees from unclustered sequences. Altogether, the trees have about 112,000 nodes, of which almost 10,000 are associated with a pattern. From the 2614 different EC-numbers in Swiss-Prot Release 57.6, 2589 (99%) are represented by at least one pattern. Out of the remaining 25 EC numbers, 21 are missing because they are only present in "putative" sequences, the other four EC numbers could not be represented in valid patterns. The smallest patterns cover nine positions, while the largest one spans as much as 5104 positions. The average pattern length is 224 positions.

We have quantified the number of enzymes for each enzyme class, subclass, and sub-subclass; and the number of trees they are clustered. The ratio sequences/trees reflects the average number of enzymes per tree and indicates the "variability" within an EC class. A high number of sequences in a few trees mean obviously low variability, while the opposite is true if the number of sequences and trees is almost equal. Table 2 shows the ten enzyme sub-subclasses with the highest and the lowest variability in this Swiss-Prot version, respectively. We have investigated the sequence lengths in the enzyme class with the highest variability, EC "3.1.21.4". There are 94 such sequences in our database, the shortest one being 157 positions and the longest one being 998 positions long. The average length is 309.6 positions, with a high standard deviation of 108.3 positions. This shows the high variability.

**Table 2:** Enzyme classes with extreme clustering properties

**EC No**.	Trees	Sequences	Seqs/Trees
3.1.21.4	73	94	1,29
3.1.6.1	5	11	2,20
1.2.7.7	6	15	2,50
3.2.1.37	6	15	2,50
5.4.99.5	7	18	2,57
1.1.99.-	5	13	2,60
2.1.1.113	8	21	2,63
3.2.1.73	5	14	2,80
3.2.1.55	9	26	2,89
1.6.99.-	5	15	3,00
2.4.1.198	8	24	3,00
3.4.16.4	6	18	3,00
4.2.1.75	5	15	3,00
4.2.2.10	4	12	3,00
**EC No**.	**Trees**	**Sequences**	**Seqs/Trees**
2.8.1.8	1	389	389,00
2.1.2.3	1	392	392,00
1.1.1.267	1	393	393,00
4.1.1.37	1	426	426,00
6.1.1.10	1	436	436,00
2.2.1.7	1	440	440,00
2.6.1.9	1	446	446,00
2.5.1.7	1	458	458,00
2.1.2.11	1	468	468,00
4.2.1.19	1	472	472,00
4.2.1.9	1	496	496,00

Table 2 displays 20 enzyme classes with extreme clustering properties. Sequences within the upper ten enzyme classes have low sequence similarity and therefore cluster in multiple trees, whereas the sequences in the lower ten enzyme classes are so similar that they cluster in only one tree.

### Implementation and runtime, memory requirements

BrEPS was implemented in Python, with MySQL as the database management system. The computation of the BLAST (NCBI, 2.2.19+) and ClustalW (1.82) alignments, as well as the verification step were parallelized to some degree. With a current Swiss-Prot version as input data, the complete BrEPS database needs about 10 GB of storage and takes less than one week to compute (with about 12 parallel processes on a 2007 compute cluster with AMD Opteron CPUs). The peak amount of main memory is needed in the clustering step, between four and eight GB.

## Discussion

The aim of the project was to develop a method to extract function-specific sequence-patterns from enzyme sequences that can be updated frequently in a fully automatic manner. The method combines the use of SwissProt-annotated sequences, the calculation of sequence-relatedness by BLAST, the construction of clusters by complete-linkage clustering and multiple alignment by ClustalW.

We are aware that some of the components we used could be modified. However, the use of an alternative to **BLAST **would not have changed the result significantly, as we are not looking for remote relationships. BLAST is more than suitable for the levels of similarity we are dealing with. The use of the clustering approach, though, has a strong impact on the result. We have chosen complete-linkage clustering, because it is very strict and provides clean clusters. This is a required property in our setup, because we do not use a dedicated domain detection protocol in BrEPS. The use of single-linkage clustering, or to less extent, UPGMA or average-linkage clustering, would lead to clusters that could contain two unrelated sequences A and B if they are connected by a multidomain sequence A-B. The strictness of the Complete Linkage clustering leads to clusters that remain quite „clean" in terms of domain composition as long as clusters are defined by low E-Values. Its only drawback may be that - because of its strictness - CL may compute more clusters than necessary and some sequences can remain unclustered. The former just leads to a few more patterns, and we take care of the latter by treating these sequences as described in the Results Section. The strictly defined clusters should be a good environment for an established and widely used progressive multiple alignment method like **ClustalW**. Of course, there may be suboptimal cases where a computed pattern is shorter than it could be. In theory, it is even possible that a multiple alignment contains a conserved position that is not biologically true, i.e. the aligned characters are not homologous. This would lead to a pattern that is not working as desired, maybe even missing its input sequences. We do however take care of these risks by filtering out short patterns, and by the verification step. We also check that all patterns recognize their input sequences. Since most of our patterns are specific, ClustalW is a good choice - and much faster than simultaneous approaches. As for the source database we are using, the **SwissProt **part of the UniprotKB [20] is the only high-quality annotated large-scale sequence database worldwide, so there was no choice here.

To evaluate the quality of BrEPS, a comparison of pattern-predicted enzyme functions with proven functions is essential. In the following sections, we describe and discuss our experiments, which involve a comparison with another automatic method for the functional annotation of enzymes.

### The standards of truth

Evaluation procedures require a standard of truth (SOT) to rate the prediction quality of the methods under evaluation. In metabolomics, a perfect standard of truth is an experimentally validated set of EC numbers representing the biochemical "toolbox" of a given organism. Even for *Escherichia coli*, such a SOT does not exist.

The PRIAM [13] authors used the Swiss-Prot annotation as SOT to evaluate their approach. Because we already used this annotation in creating the BrEPS patterns, we chose another, independent source of data to serve as SOT, one that did not involve sequence comparison in its making. The annotations in BRENDA [17] are manually extracted from experimental, published work; the AMENDA predictions are extracted from PubMed abstracts by text mining procedures [17]. We believe the BRENDA annotation to be correct in terms of specificity, i.e. every EC number that is predicted to occur in a given microorganism is really present. Unfortunately, there are rather few experimental proven enzyme functions known for many microorganisms, often only about 200-300 EC numbers are available. AMENDA usually contains about twice as many EC numbers for a given organism (the BRENDA annotations being a subset), but some of the additional predictions may be wrong. For these reasons, BRENDA serves as our "strict" standard of truth, while AMENDA serves as our "loose" SOT. Both SOTs are however incomplete, we are therefore unable to assert the number of false positives with confidence and prefer to call this experiment a "comparison of BrEPS and PRIAM" instead of an "evaluation".

### BrEPS A and B

Similar to the concept of a strict and a loose SOT described above, we define two sets of BrEPS patterns as well. The smaller BrEPS set with higher quality is called "BrEPS A" and is a subset of the more loosely defined "BrEPS B". The sets are constrained by different specificity thresholds and by the EC content of each pattern:

BrEPS A: This set contains patterns with a specificity of 1.0 (100%), i.e. patterns that produced no false positive hits in their verification. If a member of BrEPS A has more than one EC number, all EC numbers must be in the same sub-subclass (e.g., 1.1.1.*). Single, unresolved EC numbers ("1.1.1.-") are not allowed.

BrEPS B: In addition to the BrEPS A patterns, this set contains also patterns that carry EC numbers from multiple sub-subclasses. These patterns may be less specific, requiring only a specificity of 0.75 or more.

BrEPS A contains 7051 patterns, BrEPS B contains 9727 patterns. Only 125 of the additional 2676 patterns in BrEPS B have a lower specificity than 1.0, the other ones have more than one EC sub-subclass.

### Comparison to other sequence-based enzyme function predictions

A comprehensive evaluation of several methods is somewhat out of scope in this work. We compare BrEPS to PRIAM. BrEPS and PRIAM are automatic and unsupervised methods specialized on enzymes. The ProSite patterns, on the other hand, were not generated in an automatic and unsupervised way. PRATT and CASTOR are unsupervised methods for pattern extraction, they do however rely on sets of related sequences. These methods lack the similarity search/clustering part of BrEPS and PRIAM and are therefore unable to process the enzymes in the UniProt automatically.

We computed the strict and loose SOTs of five important microorganisms: *Corynebacterium glutamicum*, *Escherichia coli*, *Pseudomonas aeruginosa PAO1*, *Sulfolobus solfataricus*, and *Thermus thermophilus*, see Table 3. For each organism, we compare all its protein sequences to the BrEPS patterns and the PRIAM profiles. The PRIAM profiles we use are from the "gene-oriented release" of PRIAM (June 2009). They are better suited to annotate individual genes, instead of complete genomes. The search is made with RPSBLAST [21], the results are filtered by E-Value thresholds provided in the PRIAM file "profile_infos.txt". This file contains several columns of data per PRIAM-profile. We used the E-Values in the 4^th ^column in our comparison, because they provide the "best compromise between sensitivity and specificity" [22].

**Table 3:** Microorganisms used in comparing BrEPS to PRIAM

Species	NCBI Accession	SOT size	
		Strict	Loose
*Corynebacterium glutamicum*	*NC_003450*	97	273
*Escherichia coli*	*NC_000913*	1034	2476
*Pseudomonas aeruginosa PAO1*	*NC_002516*	368	742
*Sulfolobus solfataricus*	*NC_002754*	119	208
*Thermus thermophilus*	*NC_005838*	142	284

Table 3 shows the five microorganisms (with their NCBI Accession IDs) that were used to compare BrEPS and PRIAM. The size of the "Strict" and "Loose" standards of truth (SOT) we used is displayed on the right.

If either a BrEPS pattern or PRIAM profile matches a gene of the five micro-organisms, we compare the EC number(s) of the profile/pattern to the EC numbers in the SOT. If one of the EC numbers in the SOT is matched by one of the methods, we count a true positive hit (TP). Every EC number can only be identified once, additional hits to the same EC number are ignored. We also define a second case: If an EC number from BrEPS or PRIAM differs from an EC number in the SOT only at the fourth position, we count it as "sub-subfamily hit". Each sub-subfamily is only be counted once. Aside from an interesting insight, this also allows us to deal with unresolved EC numbers in BrEPS patterns, e.g. "1.1.1.-", where a decision would be difficult otherwise. The results of the comparison are shown in Table 4. They show that our approach is essentially on par with PRIAM. Even though most of the BrEPS A sensitivity values are considerably lower and many of the BrEPS B values are slightly lower than the PRIAM results, there are interesting exceptions: The *E. coli *values of BrEPS are mostly higher than those of PRIAM, and the BrEPS B results for *S. solfataricus *are also higher in conjunction with the loose SOT. The picture gets more interesting when sub-subclass hits are also allowed (Table 4 rightmost column): The strictly defined BrEPS A patterns gain less additional hits in contrast to PRIAM, the difference in sensitivity between BrEPS A and PRIAM is even more pronounced in this setting. This does however not hold for BrEPS B, its performance is similar to PRIAM, sometimes reaching even higher sensitivities. That can be explained with the properties of the BrEPS B patterns. Since they can be associated with multiple EC sub-subclasses, more sub-subclass hits are likely.

**Table 4:** Sensitivity of BrEPS and PRIAM

„Strict" SOT					
% True Positives			% TP incl. Sub-Subclass Hits		
BrEPS A	BrEPS B	PRIAM	BrEPS A	BrEPS B	PRIAM
45,4	52,6	55,7	76,3	90,7	92,8
61,7	69,8	64,5	67,3	79,3	67,9
45,9	53,0	54,1	64,1	74,5	73,6
36,1	41,2	42,0	58,0	68,9	68,1
43,7	50,0	52,8	66,9	79,6	80,3

**„Loose" SOT**					
**% True Positives**			**% TP incl. Sub-Subclass Hits**		

**BrEPS A**	**BrEPS B**	**PRIAM**	**BrEPS A**	**BrEPS B**	**PRIAM**

34,8	42,1	44,3	48,0	59,3	63,0
30,5	38,6	29,1	32,0	41,1	29,6
34,4	41,2	41,6	42,2	52,0	51,3
26,4	33,2	30,8	40,9	51,4	48,6
32,4	38,4	40,1	45,4	53,9	56,3

Table 4 shows the results of comparing BrEPS and PRIAM with two different standards of truth (SOT). Since the complete enzyme content of the five displayed microorganisms is not known, we only evaluate the sensitivity of both methods, i.e. the percentage of true positives (TP). BrEPS A is a strictly defined set of BrEPS patterns and a subset of BrEPS B, which is more loosely defined. The results are discussed in the main text.

Another interesting question is why BrEPS and PRIAM sometimes fail to predict the presence of an enzyme. We did a manual investigation to answer this question: From the five test microorganisms, we randomly picked five EC numbers that PRIAM found, but not BrEPS, and investigated the reason. In analogy, we also investigated five EC numbers where BrEPS scored a True Positive hit, but not PRIAM.

Our manual investigation of five cases that BrEPS revealed some of the reasons why PRIAM is slightly more sensitive than BrEPS. The main reason is that PRIAM may access more sequence data than BrEPS, because we do not allow "probable" or "putative" enzymes in creating the BrEPS patterns. Even though the annotation of most "putative" Swiss-Prot sequences will probably be correct, we do prefer to use only sequences with maximal credibility. This results in highly specific patterns that produce high-quality annotations - if the patterns do indeed match the query. The high specificity of our patterns reached at the expense of flexibility is the principal trade-off of our approach: On one hand, a single point mutation on a gene, at a position covered by "the right" pattern, is enough to make it impossible to find it. On the other hand, a few changes may also effect the loss of its catalytic activity [2], so specificity is a must in the reconstruction of metabolic networks. We are able to compensate the lack of flexibility to some degree, by computing an average of two patterns per tree. A comprehensive compilation of the BrEPS misses is also attached as additional file 1.

The five cases where PRIAM missed an enzyme also lead to an interesting insight, even though we were not able to analyze the PRIAM profiles (PSSMs) in detail, because of the file format. In all five cases the reason was the same: The suggested E-Value thresholds were too strict. For example, EC number 4.2.1.36 was missing in *T. thermophilus*. EC number 4.2.1.36 is represented by one PRIAM profile, "PRI000625". The suggested E-Value threshold of this profile is 7·10^-148^, which RPSblast missed by less than two orders of magnitude; scoring an E-Value of 2·10^-146^. The difference between the suggested threshold and the RPSblast score was less severe in the other four cases we investigated. For instance, PRIAM missed EC number 2.6.1.17 in *C. glutamicum*. The required threshold of profile PRI001252 was 1·10^-211^, while RPSblast scored only 5·10^-81.^

Finally, we investigated whether both methods find the same or different EC numbers. We have therefore analyzed the unique and common contributions of true positive EC numbers that both methods provided, see Table 5. In summary, PRIAM has a slight advantage in sensitivity. However, the results in Table 5 show that roughly 10% of the EC numbers that BrEPS found were uniquely found by BrEPS. It therefore makes sense to use BrEPS in addition to other annotation approaches, as our group does.

**Table 5:** Contribution of true positive EC numbers by BrEPS and PRIAM

Species	BrEPS A	PRIAM	Both	BrEPS B	PRIAM	Both
*C. glutamicum*	5,3	24,6	70,2	8,5	13,6	78,0
*E. coli*	5,0	9,7	85,3	8,6	1,2	90,1
*P. aeruginosa*	9,5	23,6	66,8	12,3	14,5	73,1
*S. solfataricus*	7,4	20,4	72,2	12,3	15,8	71,9
*T. thermophilus*	6,3	25,0	68,8	9,6	14,5	75,9

	**% EC numbers contributed in „Loose" SOT**					
**Species**	**BrEPS A**	**PRIAM**	**Both**	**BrEPS B**	**PRIAM**	**Both**

*C. glutamicum*	5,5	25,8	68,8	12,9	17,3	69,8
*E. coli*	14,5	10,5	75,1	25,9	1,7	72,3
*P. aeruginosa*	10,4	26,1	63,5	15,8	16,6	67,6
*S. solfataricus*	9,9	22,5	67,6	22,0	15,9	62,2
*T. thermophilus*	9,5	27,8	62,7	13,6	17,4	68,9

Table 5 shows the percentage of unique and common EC numbers that BrEPS and PRIAM contributed to the joint set of all true positive hits. BrEPS contributed between 5% and 15% unique hits, while PRIAM contributed in general between 10% and 25% unique hits; except for the *E. coli *rows in the rightmost column of Table 2, where PRIAM reached less than 2%.

## Conclusions

We have designed and implemented an automatic protocol "BrEPS" to aid in searching a given genome for enzymes, or verifying the presence of predicted enzymes. BrEPS basically computes sequence patterns of enzymes from Swiss-Prot that have been clustered at different levels of sequence similarity. In a final, iterative verification step, we ensure that the patterns are specific and cover at least a certain percentage of Swiss-Prot enzymes.

The verification results in Figure 2 showed that the quality of the BrEPS patterns is always high if they span at least about 20 sequence positions. The occasional FP outliers (e.g., around a pattern length of 50 positions in Figure 2) could also indicate mis-annotated enzymes instead of being real FPs.

A comparison with PRIAM, another method for the functional annotation of enzymes, showed that BrEPS is on average less sensitive than PRIAM. One of the main reasons is that we discard the "putative" input sequences that PRIAM is using. Nevertheless, the annotation of the *E. coli *genome showed an example where BrEPS was able to outperform PRIAM. Depending on the organism and the pattern set we used, between 5% and 25% of the joint set of EC numbers found by both methods were unique to BrEPS. We therefore conclude that BrEPS may be beneficial in combination with other approaches for functional annotation. Its main advantages are that the protocol runs automatic and unsupervised. High pattern specificity as usual is connected to a smaller sensitivity; in addition, our method relies to some degree on alignment quality, which cannot always be guaranteed.

Even though we use the really conservative Complete Linkage clustering, a future improvement of BrEPS could be to implement a domain detection protocol.

## Availability and Requirements

BrEPS will finally be integrated into other projects of our group and the patterns will be integrated into the BRENDA website. We have set up a preliminary website ("Broli", short for "BrEPS Online") at http://breps.tu-bs.de. It allows the user to submit either an EC number or a protein sequence. If the input is an EC number, Broli will retrieve all patterns associated with that EC number. If the input is a protein sequence, Broli will search this sequence with all BrEPS patterns and display the matching patterns in a condensed way. The complete information available for interesting patterns can be shown by toggling the corresponding check boxes and clicking "submit" again. The user can also choose to display all patterns of a given tree by clicking on one of the links in the "Tree" column.

The source code is available on Request (by Email to DS) for non-commercial, academic use only. In addition the calculated enzyme-specific patterns will be implemented in and made available in the next update of the BRENDA enzyme information system.

## Authors' contributions

CadS devised the first part of BrEPS in his PhD thesis [23], largely corresponding to the "Preprocessing" paragraph in the Results section. AW devised the second part of the method during his PhD thesis [24], corresponding to the "Pattern Construction" and "Pattern Verification" sections in the Results section. CB integrated both projects into one protocol and improved some of the existing work. He also implemented the Complete Linkage clustering and parallelized a part of the code to run on our compute cluster. DS had the initial idea; he supervised and coordinated the work of the other authors. CB, AW, and DS contributed to this paper. All authors have read and approved the final manuscript.

## Supplementary Material

Additional file 1 **Manual analysis of five EC numbers that BrEPS could not detect**. The attached .pdf file shows a table that illustrates our manual analysis of five EC numbers from five microorganisms that were found by PRIAM with confidence, but not by BrEPS. It also contains some notes on our analysis process. Click here for file
