# Supplementary material for: BrEPS: a flexible and automatic protocol to compute enzyme-specific sequence profiles for functional annotation
Source: BMC Bioinformatics. 2010 Dec 1;11:589. doi: 10.1186/1471-2105-11-589 (PMC3009691; doi:10.1186/1471-2105-11-589)
Supplement: Additional file 1 — Manual analysis of five EC numbers that BrEPS could not detect. The attached .pdf file shows a table that illustrates our manual analysis of five EC numbers from five microorganisms that were found by PRIAM with confidence, but not by BrEPS. It also contains some notes on our analysis process. [file 1471-2105-11-589-S1.PDF]

Case	Data and description
1	**EC-Number:** 1.1.1.169 (2-dehydropantoate 2-reductase)<br>**Species:** *C. glutamicum*<br>**Missed target protein; source:** "CG_Q8NRG0"; TrEMBL<br>**Description:** The BrEPS pattern was too specific to hit the target sequence. Being a TrEMBL sequence, the target sequence is not in our database and its properties are therefore missing in our pattern. More important, the „Enzyme" database that provides the sequence collections for PRIAM also contains "putative" Swiss-Prot sequences that BrEPS excludes. The corresponding PRIAM profile represents more information than the BrEPS pattern and is therefore less specific, hence it hit the target.
2	**EC-Number:** 2.4.1.7 (Sucrose phosphorylase)<br>**Species:** *E. coli*<br>**Missed target protein; source:** "P76041"; Swiss-Prot<br>**Description:** The BrEPS pattern was again too specific too match the target sequence. The target is a "putative" Swiss-Prot entry that is therefore not present in our pattern. As in case 1, the PRIAM profile contains additional information that is missing in BrEPS.
3	**EC-Number:** 3.5.3.4 (Allantoicase)<br>**Species:** *P. aeruginosa*<br>**Missed target protein; source:** "Q9I3J8"; Swiss-Prot<br>**Description:** The BrEPS pattern was too specific and/or suboptimal. The target sequence is annotated as "probable allantoicase" in Swiss-Prot. The PRIAM profile was computed from 41 Swiss-Prot sequences, of which 35 are annotated as "probable". The remaining six sequences are the data source of the BrEPS pattern. They are are not very similar and cluster at an E-Value of 10^{-6}. Their multiple alignment looks suboptimal, which, together with the small amount of sequence information, explains why the BrEPS pattern did not match the sequence.
4	**EC-Number:** 5.99.1.2 (DNA topoisomerase I)<br>**Species:** *S. solfataricus*<br>**Missed target protein; source:** "Q97ZJ8"; TrEMBL<br>**Description:** The BrEPS pattern did not match the query for rather complex reasons. The TrEMBL target is probably a "DNA topoisomerase I". Many (if not all) species have this enzyme and divergent evolution has lead to many variations of it. To cover the sequence space of 5.99.1.2, PRIAM uses five profiles. BrEPS has 11 patterns in ten trees at its disposal, with E-Values ranging from 10^{-180} to 10^{-9}. Some patterns do not match because their input sequences are just not similar to the query, while others are too specific. The exact circumstances are even harder to determine because two of the BrEPS patterns carry multiple EC numbers – which raises questions about alignment quality.
5	**EC-Number:** 6.1.1.4 (Leucine--tRNA ligase)<br>**Species:** *T. thermophilus*<br>**Missed target protein; source:** "Q7SIE4"; TrEMBL<br>**Description:** The TrEMBL target is not part of a BrEPS pattern. There are 679 Swiss-Prot sequences with the given EC number in our database, they clustered into 16 patterns, of which 14 had an E-Value better than 10^{-180}, i.e. 0. This means that these sequences readily cluster into several groups, but have little variation within each cluster. Therefore most of our patterns were too specific to hit.
